# Proteomic and Biochemical Changes during Senescence of *Phalaenopsis* ‘Red Dragon’ Petals

**DOI:** 10.3390/ijms19051317

**Published:** 2018-04-28

**Authors:** Cong Chen, Lanting Zeng, Qingsheng Ye

**Affiliations:** Guangdong Provincial Key Lab of Biotechnology for Plant Development, School of Life Sciences, South China Normal University, Guangzhou 510631, China; parker_cc@163.com (C.C.); zenglanting@scbg.ac.cn (L.Z.)

**Keywords:** *Phalaenopsis*, petal, pollination, senescence, 2-DE, ROS

## Abstract

*Phalaenopsis* flowers are some of the most popular ornamental flowers in the world. For most ornamental plants, petal longevity determines postharvest quality and garden performance. Therefore, it is important to have insight into the senescence mechanism of *Phalaenopsis*. In the present study, a proteomic approach combined with ultrastructural observation and activity analysis of antioxidant enzymes was used to profile the molecular and biochemical changes during pollination-induced petal senescence in *Phalaenopsis* “Red Dragon”. Petals appeared to be visibly wilting at 24 h after pollination, accompanied by the mass degradation of macromolecules and organelles during senescence. In addition, 48 protein spots with significant differences in abundance were found by two-dimensional electrophoresis (2-DE) and subjected to matrix-assisted laser desorption/ionization time of flight mass spectrometry (MALDI-TOF/TOF-MS). There were 42 protein spots successfully identified and homologous to known functional protein species involved in key biological processes, including antioxidant pathways, stress response, protein metabolism, cell wall component metabolism, energy metabolism, cell structure, and signal transduction. The activity of all reactive oxygen species (ROS)-scavenging enzymes was increased, keeping the content of ROS at a low level at the early stage of senescence. These results suggest that two processes, a counteraction against increased levels of ROS and the degradation of cellular constituents for maintaining nutrient recycling, are activated during pollination-induced petal senescence in *Phalaenopsis*. The information provides a basis for understanding the mechanism regulating petal senescence and prolonging the florescence of *Phalaenopsis*.

## 1. Introduction

*Phalaenopsis*, named for its butterfly-like flowers, is known as the “queen of the orchids” for its graceful shape and colorful flowers. *Phalaenopsis* flowers are among the most popular ornamental flowers in the world and have high economic value [1]. For most ornamental plants, petal longevity determines postharvest quality and garden performance [2], so it is essential to have insight into the mechanism regulating petal senescence.

The longevity of *Phalaenopsis* petals is under tight developmental control for up to 3 months [3], so it is difficult to understand the mechanism of regulating petal senescence, which can be affected by many environmental factors. However, its petal senescence can be almost synchronized with pollination, and detectable signs of senescence can occur within one day [4]. Petal senescence and shedding are the earliest and most obvious changes induced by pollination [5]. Therefore, pollination treatment provides a quick and efficient approach for the study of *Phalaenopsis* petal senescence.

Petal senescence is accompanied by a series of ultrastructural and physiological-biochemical changes that form the last stage of flower development [6,7]. The maintenance of petals is costly in terms of water loss and metabolic energy; therefore, cellular constituents such as macromolecules and organelles are degraded so that nutrients can be recycled for reallocation to developing tissues [8]. As a conserved system, autophagy supports the recycling function, appearing to play a crucial role in degradation during petal senescence [6]. Reactive oxygen species (ROS) play a key role in the regulation of many developmental processes, including senescence, as well as in plant responses to biotic and abiotic stresses [9]. The activity of ROS-scavenging enzymes tends to increase during senescence [10]. Simultaneously, there is a concomitant increase in the level of malondialdehyde (MDA), which acts as an indicator of lipid peroxidation [11].

The combination of two-dimensional electrophoresis (2-DE) and matrix-assisted laser desorption/ionization time of flight mass spectrometry (MALDI-TOF/TOF-MS) analyses allows research on the plant at the post-transcriptional level; i.e., the protein level. This combination has been used to study not only plant developmental processes [12,13], but also stress responses [14,15]. These proteomic studies have provided unique insight into the role of post-translational modifications regulating and executing plant development and stress response. Coincidentally, senescence is also controlled at the post-transcriptional level [16]. Therefore, the proteomic approach is feasible for investigating petal senescence.

The regulation of gene expression occurs at the protein and the transcript levels. Therefore, the mechanism regulating petal senescence in *Phalaenopsis* is complex and unclear. In the present study, we report on the first proteomic characterization of *Phalaenopsis* petal senescence, combined with ultrastructural observation and physiological-biochemical analysis. The aim of this study is to elucidate the dynamic changes that occur at the molecular and biochemical levels during petal senescence.

## 2. Results

### 2.1. Ultrastructural Changes of Organelles in Senescing Phalaenopsis Petals

**Figure 1:**
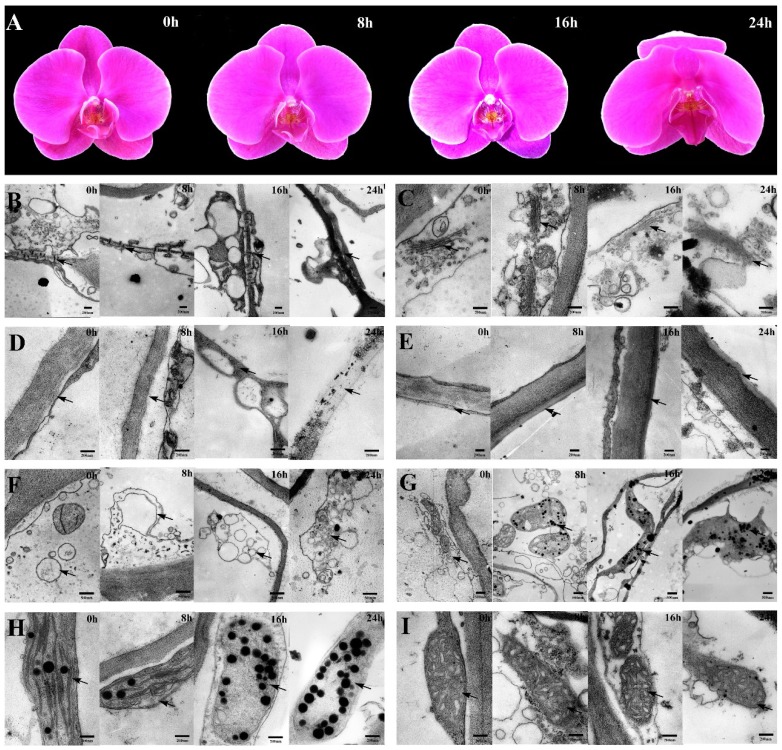
(**A**) Morphological changes of *Phalaenopsis* petal during pollination-induced senescence; (**B**–**I**) Ultrastructural changes of *Phalaenopsis* petal during pollination-induced senescence. (**B**) Plasmodesmata (×20,000); (**C**) Golgi apparatus (×40,000); (**D**) mesophyll cell wall (×40,000); (**E**) epidermal cell wall (×25,000); (**F**) vacuole (×15,000); (**G**) osmiophilic granule (×10,000); (**H**) chloroplast (×40,000); (**I**) mitochondria (×40,000). Corresponding structures in each figure are indicated by arrows.

In our experiment, *Phalaenopsis* showed 2–3 months’ flower longevity in natural conditions. By contrast, pollination dramatically accelerated the senescence of petals, and flowers visibly wilted at 24 h after pollination (Figure 1A).

The ultrastructural changes in *Phalaenopsis* petals during pollination-induced senescence are shown in Figure 1B–I. The plasmodesma was partially closed at 8 h and completely closed at 16 h in pollinated flowers (Figure 1B). By contrast, the Golgi bodies were almost intact, except for the local structural distortion exhibited in Figure 1C. Mesophyll cell walls showed plasmolysis at 8 h and were severely degraded at 24 h, whereas epidermis cell walls hardly changed within 24 h after pollination (Figure 1D,E). The vacuoles appeared to have lost membrane integrity as senescence progressed (Figure 1F). Cells containing vacuoles of various sizes were observed, and the vacuoles contained many vesicles and granules in pollinated flowers at 16 h. At the same time, numerous osmiophilic granules were revealed (Figure 1G). For chloroplasts, the ongoing increase in the number of osmiophilic granules was concomitant with the loss of a considerable portion of thylakoids and double-layer membrane structures (Figure 1H). Figure 1I shows that the mitochondria had swelled and the cristae had degraded to a lesser extent in the cells 24 h after pollination; however, the mitochondria were nearly intact overall. The nucleus remained until a late stage of senescence, although several nuclear ultrastructural changes were observed at 24 h, such as shrinkage trait, loss of ellipticity, and blebbing. Peroxisomes were not present in *Phalaenopsis* petal cells during pollination-induced senescence.

### 2.2. Protein Profiling and Analysis of Protein Species Changes

Protein yield obtained from *Phalaenopsis* petals after phenol-based extraction was evaluated. Protein yields were the same among petals at four time points, in the range of 6.03 ± 0.94 mg/g of fresh weight (Table 1).

**Figure 2:**
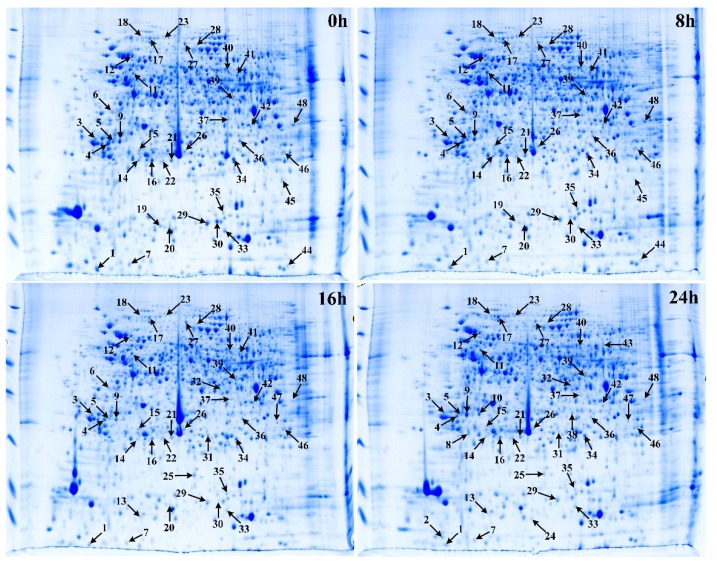
Representative two-dimensional electrophoresis (2-DE) gels of *Phalaenopsis* petal proteomic variation during pollination-induced senescence. The protein spots were separated on immobilized pH gradient (IPG) dry strips (24 cm in length, pH 3–10 nonlinear gradient (NL)). The numbers on the left in the images indicate the corresponding protein spots listed in Table 2.

To analyze the variation of protein species in pollination-induced senescing *Phalaenopsis* petals, the differentially regulated protein spots were separated by 2-DE and identified with MALDI-TOF/TOF-MS. In 2-DE of the petal protein extract, more than 1000 protein spots per gel were consistently observed in all replicates, with molecular weight (MW) and isoelectric point (pI) ranging from <14 kDa to 115 kDa and 4 to 9, respectively (Figure 2). As shown in Table 1, the average proteomic maps were 1069 ± 92, 1061 ± 203, 1056 ± 80, and 1014 ± 8 for 0, 8, 16, and 24 h, respectively. The mean coefficient of variance (CV) for all of these samples was 9.12%. Based on the criteria for protein spot detection, 48 protein spots with significant differences in abundance were detected in response to pollination and 42 protein spots were confidently identified according to the databases; six protein spots (3, 21, 22, 27, 28, and 45) could not be identified conclusively.

### 2.3. Functional Classification of Differentially Regulated Protein Species

Among 42 protein spots, during petal senescence, 17 protein spots (2, 8, 10, 13, 17, 18, 23, 24, 25, 31,32, 37, 38, 39, 43, 46, and 47) were upregulated and 25 protein spots (1, 4, 5, 6, 7, 9, 11, 12, 14, 15, 16, 19, 20, 26, 29, 30, 33, 34, 35, 36, 40, 41, 42, 44, and 48) were downregulated at one or more time points compared with 0 h. The 2-DE map acquired from 0 h petals was taken as the control, and qualitative differences in spot intensity between the control (0 h) and the pollination treatment (8, 16, or 24 h) were found and are displayed in Table 1. The number of differentially regulated protein spots was obtained from the changes of 42 spots’ abundance. As shown in Table 1, by 8 h after pollination, one protein spot was upregulated and 14 protein spots were downregulated in petals by greater than 1.5-fold (*p* < 0.05). There were 5 protein spots upregulated and 22 protein spots downregulated at 16 h (*p* < 0.05). At 24 h after pollination, the numbers of upregulated and downregulated protein spots were 16 and 23, respectively.

**Figure 3:**
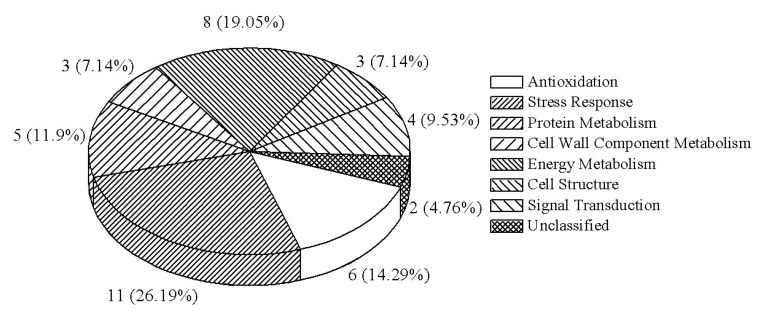
Functional categorization of the differentially regulated protein species in *Phalaenopsis* petals during pollination-induced senescence.

All proteins identified and the correspondence between a given spot number and the assigned protein species are detailed in Table 2. According to the Gene Ontology (GO) and Kyoto Encyclopedia of Genes and Genomes (KEGG) annotations, as well as the related references, the 42 protein species were classified into eight groups (Figure 3): six (14.3%) were involved in the antioxidant pathway, 11 (26.2%) in the stress process, five (11.9%) in protein metabolism, three (7.1%) in cell wall component metabolism, eight (19.1%) in energy metabolism, three (7.1%) in cell structure, four (9.5%) in signal transduction, and the remaining two (4.8%) were not classified.

In this study, protein species were identified as being associated with pollination-induced senescence, improving our knowledge of how senescence progresses in *Phalaenopsis* petals. Further studies are needed to characterize the mechanism of pollination-induced senescence in more detail.

### 2.4. Changes in Antioxidant Enzymes and MDA

**Figure 4:**
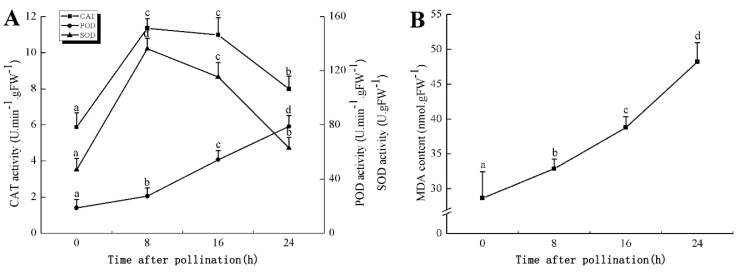
(**A**) Changes of superoxide dismutase (SOD), catalase (CAT), and peroxidase (POD) activity in *Phalaenopsis* petal during pollination-induced senescence; (**B**) Change of malondialdehyde (MDA) content in *Phalaenopsis* petal during pollination-induced senescence. Values are presented as means ± SD. Means distinguished with different letters are significantly different from each other (*p* ≤ 0.05).

In the proteomic study described above, it was found that thioredoxin-dependent peroxidase (POD, spot 10) was upregulated during petal senescence. However, the relationship between POD regulation and activity changes was unknown. Hence, the activity of three ROS-scavenging enzymes, superoxide dismutase (SOD), catalase (CAT), and peroxidase (POD), was measured. During petal senescence, the activity of POD continuously increased (Figure 4A), which was consistent with the proteomic result. The activity of SOD and CAT changed simultaneously with POD, but noticeably decreased after 8 h in pollinated flowers (Figure 4A). Additionally, malondialdehyde (MDA) levels content increased considerably in the senescing petals of pollinated flowers compared with the control samples (0 h) (Figure 4B).

## 3. Discussion

### 3.1. Ultrastructural Analysis

Usually, one of the earliest ultrastructural changes is the closure of plasmodesmata, which, when open, allow the transfer of relatively small molecules such as sugars, hormones, and RNA molecules between neighboring cells [17]. In the present study, the plasmodesmata indeed showed significant changes at 8 h (Figure 1B). In several species, such as *Alstroemeria* and *Sandersonia*, petal mesophyll cells were found to die considerably earlier than epidermis cells [18,19], and this phenomenon was also shown in senescing *Phalaenopsis* petals. In our experiment, petal cells in *Phalaenopsis* exhibited a decrease in vacuolar size and an increase in the number of small vacuoles because of the zoned phenomenon of vacuoles during senescence (Figure 1F), which is in line with other plants such as petunia [6]. The appearance of osmiophilic granules is a typical organelle alteration during aging [20]. Accordingly, in this study the increased number of osmiophilic granules demonstrated that organelles such as chloroplasts and mitochondria also changed in senescing petals (Figure 1G,H). Visible external senescing syndrome was the outcome of vast ultrastructural changes.

Collectively, the data indicate that petal senescence was accompanied by the mass degradation of macromolecules and organelles, and the ultrastructural changes to the structures and organelles were typical features of programmed cell death (PCD). From Figure 1, it can be seen that visible morphological changes of pollinated petals occurred at 24 h, whereas ultrastructure exhibited significant changes after 16 h, indicating that the ultrastructural change of petals was the structural basis of morphological changes. The protein species that play important roles in petal senescence are differentially regulated in the early stage, before visible morphological changes occur. Therefore, we selected four time points within 24 h (0, 8, 16, and 24 h) where the petals did not show dramatic wilting.

### 3.2. Protein Function Analysis

In this study, a number of functional protein species participated in regulation, as evident from their differential regulation in non-senescing and senescing petals; these protein species were likely to be involved in various pathways, including antioxidant pathways, stress response, protein metabolism, cell wall component metabolism, energy metabolism, cell structure, and signal transduction.

#### 3.2.1. Antioxidant Enzymes

Plants have evolved complex regulatory mechanisms to prevent cellular injury through regulating destructive ROS so that they are present at steady-state levels [21]. In our study, several protein species involved in antioxidant pathways, including thioredoxin-dependent peroxidase (POD, spot 10), thioredoxin H-type (TRX, spot 7), glutathione *S*-transferase (GST, spots 14, 16, and 34), and NAD(P)-linked oxidoreductase (spot 41), were found to be differentially regulated during petal senescence. All of these protein species are components of a pathway that is activated by the plants themselves to remove ROS.

POD is important for scavenging ROS and for protecting the cellular membrane from peroxidation [22]. Usually, the peroxidase family members serve as detoxifying enzymes and perform oxidation reactions to remove toxic reductants [23]. Indeed, our results showed that the abundance of POD (spot 10) increased during petal senescence, which was consistent with the measured POD activity (Figure 4A). TRX, which functions as a regulator of apoplastic ROS, maintains redox balance via thiol-disulfide exchange reactions and plays a critical role in responding to ROS-induced cellular damage [24]. Another detoxifying enzyme, GST, can protect a senescing cell from lipid hydroperoxides prior to cell death. An oxidative burst and GST induction are usually used as markers of induction of the defense response [25]. As our results show, the abundances of TRX (spot 7) and GST (spots 14, 16, and 34) were very high in early senescence but decreased at 16 h, possibly because there were some circadian or diurnal effects on these patterns. Diurnal redox behaviors of these two enzymes were strictly linked to light intensity and the mRNA of TRX and GST tended to be lower in the dark phase (16 h) [26]. Ratnayake et al. [27] found that an alternative way to increase the antioxidant status of a cell is to enhance the defense system involving cytoprotective antioxidant enzymes, including NAD(P)H: quinone oxidoreductase 1 (NQO1) [27]. However, NAD(P)-linked oxidoreductase (spot 41) was downregulated in pollinated petals after 16 h, from which it could be postulated that an oxidative burst might turn up at that time, an actual time point of senescence.

In the present study, the abundance of these enzymes after pollination indicated that petals outperformed themselves to maintain the metabolic balance of the active oxygen system, thereby postponing the senescence process. Therefore, we can conclude that enhancing the gene expression of ROS-scavenging enzymes is an effective way to prolong *Phalaenopsis* petal longevity.

#### 3.2.2. Stress Response Protein Species

Phospholipase D (PLD) hydrolyzes membrane lipids to generate phosphatidic acid (PA) and a free-head group, which destroy membranes and activate other lipid-degrading enzymes from the hydrolysis of membrane phospholipids [28]. In PLDδ-knockout *Arabidopsis*, leaf senescence was delayed because the production of PA was repressed by the attenuation of lipid degradation [29]. In this context, the upregulation of PLD (spot 31) was exclusively detected in 2-DE gels, perhaps demonstrating that petal senescence was accompanied by increased phospholipid catabolism for maximum cellular recycling [30]. Alcohol dehydrogenase (ADH) catalyzes redox reactions between acetaldehyde and ethanol, participating in plant anaerobic respiration. It is an inducible enzyme and can be activated under some adverse conditions [31]. Therefore, ADH may be crucial for regulating plant stress resistance and accommodating to adversity. Nevertheless, no similarities to these previous findings were observed in this study; the abundance of ADH (spot 42) was decreased, suggesting that the ethanol fermentation pathway might be shut down during senescence. Therefore, it is speculated that the shutdown of this pathway is one of the drivers of the decrease in reducing capacity and the increase in ROS levels. However, the relationship between this pathway and ROS levels remains unclear.

Above all, pollination-induced *Phalaenopsis* petal senescence is a very complex process. The differential regulation of protein species involved in antioxidant pathways and stress response in senescing petals might be closely related to ROS. Nonetheless, how these protein species interact with each other remains unknown.

#### 3.2.3. Protein Species Involved in Protein and Cell Wall Component Metabolism

The total protein level decreased drastically prior to visible senescence symptoms in the petals [32]. The decrease in protein levels is primarily due to an increase in degradation, as well as a decrease in synthesis [17], demonstrating that large-scale degradation occurs during senescence. Additionally, nutrient remobilization from senescent organs requires the action of a suite of degradative enzymes [25].

Cysteine protease (CP) is encoded by *SAG12*/*Cab*, which acts as a molecular indicator of leaf senescence progression [33]. Battelli et al. [34] also found that CP is responsible for most of the proteolytic activity in senescent petals [34]. Our results show that CP (spot 32) accumulated during petal senescence, which agrees with previous observations [35]. Aminopeptidase can be induced at high carbohydrate levels, which may initiate senescence and result in nitrogen remobilization [36]. In our experiment, aminopeptidase N (APN, spot 23), which might be important for nitrogen nutrient recycling, was upregulated. In short, these four protein species sufficiently supported nutrient remobilization during the senescence stage. Three protein species related to cell wall component metabolism, namely cellulose synthase (CesA, spot 9), xyloglucan endotransglucosylase/hydrolase (XTH, spot 38), and cinnamyl alcohol dehydrogenase (CAD, spot 39), were identified. CesA catalyzes the conversion of d-glucose to cellulose via β-1, 4-glycosidic bonds [37]. XTH, a primary cell-loosening enzyme, can catalyze the degradation of xyloglucan, which is the primary composition of hemicellulose [38]. An increase in XTH and a decrease in xyloglucan during petal senescence were found in previous studies [19,39]. Cellulose and hemicellulose are components of the cell wall; therefore, a decrease in these two substances may cause a bend in the shape of the flowers. In this study, the downregulation of CesA (spot 9) and upregulation of XTH (spot 38) resulted in the degradation of cell wall components, affecting the external morphology of the petals. CAD is the limiting enzyme in lignin synthesis, a secondary metabolic process [40]. Lignin provides structural rigidity for tracheophytes to stand upright and strengthen the cell walls [41]. The upregulation of XTH (spot 38) and downregulation of CAD (spot 39) were both obviously exacerbated at 16 h after pollination, suggesting that the cell wall components were broken down and degraded gradually, which was in agreement with the ultrastructural observations (Figure 1D). This phenomenon supports a viewpoint that ethylene burst (accelerating petal senescence) is started after several hours, not at 0 h, after pollination in orchids [42].

#### 3.2.4. Energy Metabolism Protein Species

Petal senescence after pollination denoted a massive increase in carbon flow through glycolysis and the tricarboxylic acid cycle (TCA), and protein spots corresponding to related enzymes (spots 8, 11, 15, 17, 18, 19, 26, and 40) were identified. Pyrophosphate-fructose 6-phosphate 1-phosphotransferase subunit β (PFP, spot 40) and triosephosphate isomerase (TPI, spots 8 and 15) are important enzymes in glycolysis, and two of these (spots 40 and 15) became downregulated once pollination occurred. One possibility for this phenomenon would be a self-defense mechanism in which low glucose metabolism contributed to sugar accumulation, sequentially inhibiting the expression of senescence-associated genes (SAGs), given that van Doorn et al. [43] proposed that sugar starvation would directly result in petal senescence, and vice versa [43]. Nonetheless, the upregulation of spot 8 might be conducive to maintaining carbon recycling during petal senescence. Dihydrolipoamide acetyltransferase (E2, spot 11) and dihydrolipoamide dehydrogenase (E3, spot 19) are components of the pyruvate dehydrogenase system, which catalyzes the conversion of pyruvate into acetyl-CoA. A decrease in the abundance of these two protein species demonstrated that the TCA cycle is downregulated during petal senescence induced by pollination, as noted in a review by van Doorn and Woltering [17]. The level of pyruvate orthophosphate dikinase (PPDK, spots 17 and 18) increased during senescence, consistent with the viewpoint that the abundance of PPDK might dramatically increase during abiotic stress, such as low-oxygen stress and water deficit [44]. A reduction in ATP synthase (ATPase, spot 26) abundance occurred, possibly because this membrane enzyme produces ATP—generated from the downregulated TCA cycle—from ADP in the presence of a proton gradient across the membrane.

#### 3.2.5. Signal Transduction Protein Species

Four protein species (spots 4, 25, 36, and 48) related to signal transduction were successfully identified. Numerous studies have demonstrated that 14-3-3 protein is a key anti-apoptotic factor that is upregulated in senescent plants and blocks apoptosis by inhibiting the activation of p38 MAPK. In addition, 14-3-3 protein binds to the apoptosis-promoting protein BAD and to forehead transcription factor FKHRL1, inhibiting the stimulation of apoptosis [45]. In this study, the upregulation of 14-3-3 protein (spot 25) perhaps demonstrated that 14-3-3 protein can postpone petal senescence by repressing the activity of apoptotic factors. GTP-binding protein (GTP) regulates many physiological processes, such as vesicular transportation, signal transduction, and cell apoptosis [46]. Casein kinase II (CKII) is a highly conserved and messenger-independent serine/threonine protein kinase with both cytosolic and nuclear localization in eukaryotic cells [47]. This enzyme is implicated in important biological processes, including apoptosis. In our study, the downregulation of GTP (spot 4) and CKII (spot 36) during petal senescence differed from the regulation changes in GTP and CKII observed in *Mangifera indica* and *Coleus blumei* [48,49], and further study is needed to obtain the satisfactory elucidation of these findings.

### 3.3. Physiological-Biochemical Analysis

The functions of ROS-scavenging enzymes in senescing petals include detoxifying ROS and preventing the accumulation of toxic substances. In this context, POD activity was higher in pollination treatment (8, 16, and 24 h) than in the control (0 h) (Figure 4A), as has been shown in other plants, such as carnation and day lily [50,51]. The activity of SOD and CAT rapidly increased in the first 8 h (Figure 4A), which was similar to previous observations in *Hemerocallis* (day lily) flowers [51], demonstrating that these enzymes provide the first line of defense against ROS. However, the overall picture shows a reduction in the activity of ROS-scavenging enzymes as senescence progresses after 8 h, with earlier decreases in the activity of SOD and CAT compared to POD, corresponding to the view proposed by Zeng et al. [50]. The levels of MDA were elevated considerably in the senescing petals of pollinated flowers (Figure 4B), in agreement with previous results [11].

In the 2-DE results, the content of POD was increased during pollination-induced senescence. There is a close relationship among POD, CAT, and SOD, which are important antioxidant systems that catabolize superoxide and hydrogen peroxide, a precursor of reactive oxidants [52,53]. Based on the 2-DE results and previous studies, we also monitored the changes in the activities of these three enzymes during petal senescence. The results showed that the activities of the antioxidant enzyme were also increased. The 2-DE and physiological-biochemical results showed that the ROS-scavenging system was activated after pollination treatment in *Phalaenopsis* petals. However, we were not able to explain why the presence of peroxisome marker enzymes was detected and no peroxisomes were highlighted with the ultrastructural analysis.

Overall, the effective ROS-scavenging system was rapidly activated once pollination occurred and maintained ROS at a controlled level, delaying the senescence process.

### 3.4. Possible Processes That Regulate Differentially Regulated Protein Species during Petal Senescence

**Figure 5:**
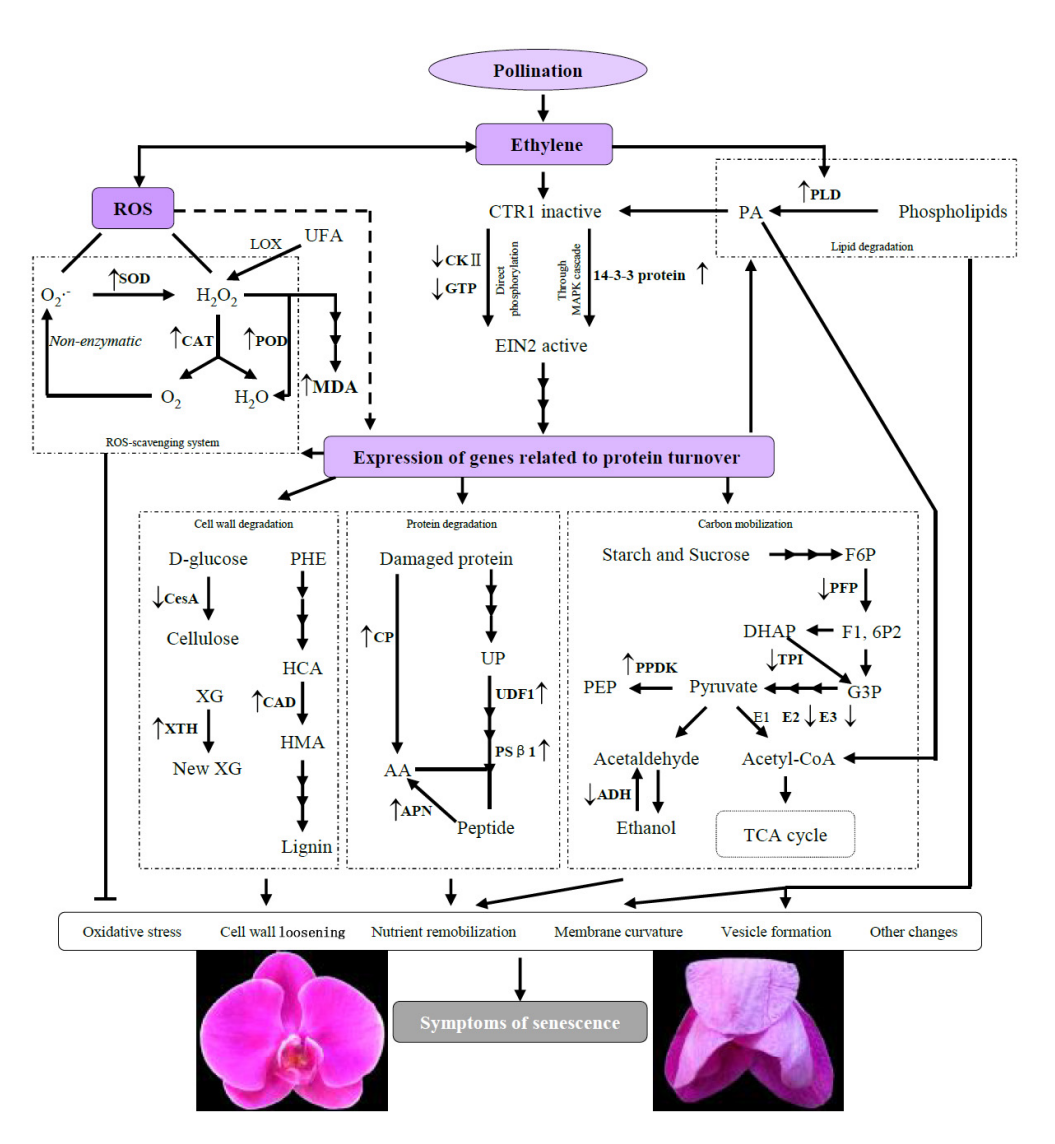
Putative functions of differentially regulated protein species during petal senescence. Identified protein species are shown in bold text. Upregulated protein species are marked with ↑, and downregulated protein species are marked with ↓. ‘Mlti-step enzymatic reaction’ is marked with 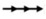. ROS, reactive oxygen species; SOD, superoxide dismutase; CAT, catalase; POD, peroxidase (spot 10); UFA, unsaturated fatty acid; LOX, lipoxygenase; MDA, malondialdehyde; CK II, casein kinase II (spot 36); GTP, GTP-binding protein (spot 4); 14-3-3 protein (spot 25); PLD, phospholipase D (spot 31); PA, phosphatidic acid; CesA, cellulose synthase (spot 9); XG, xyloglucan; XTH, xyloglucan endotransglucosylase/hydrolase (spot 38); PHE, phenylalanine; HCA, hydroxycinnamic aldehyde; CAD, cinnamyl alcohol dehydrogenase (spot 39); HMA, hydroxy cinnamic alcohol; CP, cysteine proteinase (spot 32); UP, ubiquitinated protein; UDF1, ubiquitin fusion degradation protein 1 (spot 37); PSβ1, proteasome subunit β type 1 (spot 43); AA, amino acid; APN, aminopeptidase N (spot 23); F6P, fructose-6-phosphate; PFP, pyrophosphate-fructose 6-phosphate 1-phosphotransferase (spot 40); F1,6P2, fructose-1, 6-diphosphate; DHAP, dihydroxyacetone phosphate; TPI, triosephosphate isomerase (spots 8 and 15); G3P, glyceraldehydes-3-phosphate; PPDK, pyruvate orthophosphate dikinase (spots 17 and 18); E1, pyruvate dehydrogenase; E2, dihydrolipoamide acetyltransferase (spot 11); E3, dihydrolipoyl dehydrogenase (spot 19); ADH, alcohol dehydrogenase (spot 42); TCA, tricarboxylic acid cycle.

Based on the functions of the differentially regulated protein species, a possible mechanism of petal senescence is proposed (Figure 5). Initially, pollination could trigger the upregulation of ethylene biosynthetic genes [21]. In this study, ethylene-responsive transcription factors were activated by direct phosphorylation (possibly by CK II and GTP) or an MAPK cascade (14-3-3 protein) [45]. Other transcription factors were activated by other signals, probably including PA and ROS [9,54]. An increase in ethylene production could activate PLD, which would contribute to rapid PA accumulation. PA formation could switch on downstream ethylene responses via interaction of the lipid with CTR1 [54]. PA itself could also promote membrane curvature and induce vesicle formation [55]. A burst of ROS production might activate the ROS-scavenging system during the early stage of senescence. The abundance and activity of antioxidant enzymes such as SOD, CAT, and POD were upregulated, postponing early senescence. However, in general, during petal senescence, ROS levels rose and antioxidant levels fell, resulting in oxidative stress [9]. Many other protein species involved in cell wall degradation (CesA, XTH, and CAD), protein degradation (CP, UDF1, PSβ1, and APN), and carbon mobilization (PFP, TPI, PPDK, ADH, E2, and E3) were also differentially regulated. Cell wall degradation might result in the loosening of the cell wall, as shown by the ultrastructural results; meanwhile, protein degradation and carbon mobilization supported nutrient remobilization. Overall, each of these differentially regulated protein species plays a unique and cooperative role in regulating petal senescence.

## 4. Materials and Methods

### 4.1. Plant Materials

*Phalaenopsis* “Red Dragon” plants were grown in a greenhouse at South China Normal University (Guangzhou, China). Flowers in the middle of inflorescence were selected. They were hand pollinated, using the method described by Visser et al. [56], in the morning (10:00) on the first day after flowering and were examined hourly thereafter for visible morphological changes. The petals were collected from pollinated flowers at 0, 8, 16, and 24 h. For proteomic and physiological-biochemical analysis, samples were frozen under liquid nitrogen rapidly and stored at −80 °C until required. Ten fresh petals were collected from one group (20 plants) at random and pooled together as one biological replicate of each time point. In this experiment, three biological replicates and three technical replicates of three biological replicates were conducted for proteomic analysis and physiological-biochemical analysis, respectively.

### 4.2. Observation of the Petal Ultrastructure

To visualize autophagic processes in *Phalaenopsis* petals, samples collected at each time point were observed by using transmission electron microscopy (TEM). The experimental petals of each time point were prepared from flowers that were chosen from one group (20 plants) at random. The operating steps were conducted as recommended by Shibuya et al. [6] with the following modifications [6]. The center section of each petal was selected and cut into pieces (5 mm × 2 mm). The sample pieces were fixed in 0.1 M phosphate buffer (pH 7.0) (containing 4% paraformaldehyde and 4% glutaraldehyde) at 4 °C for at least 4 h. After fixation, the sample pieces were rinsed with 0.05 M phosphate buffer, followed by post-fixation in 0.1 M phosphate buffer (pH 7.0) (containing 1% osmium tetroxide) at 4 °C for 2 h. Then, dehydrated specimens were embedded in epoxy resin. Ultrathin sections (70 nm) were made with a diamond knife using an ultramicrotome (RM 2255; Leica, Wetzlar, Germany) and placed on copper grids. Sections were stained with 2% uranyl acetate at room temperature for 15 min and rinsed with double-distilled water, followed by secondary staining with a lead-staining solution at room temperature for 3 min. Lastly, the sections were observed and photomicrographs were recorded with a transmission electron microscope (JEM-1010; JEOL, Tokyo, Japan).

### 4.3. Protein Extraction

A phenol-based extraction method was employed for protein extraction with the following modifications [57]. Frozen *Phalaenopsis* petals (1.5 g) were finely powdered in liquid nitrogen and suspended in 4 mL ice-cold extraction buffer (500 mM Tris-HCl buffer pH 8.0 containing 0.7 M sucrose, 50 mM ethylenediaminetetraacetic acid, 100 mM KCl, and 2% (*v/v*) β-Mercaptoethanol) and 4 mL water-saturated phenol (pH < 4.5) in a 10-mL centrifuge tube. The homogenate was left for 30 min and centrifuged at 19,500× *g* for 30 min at 4 °C. The upper phenolic phase was collected into a new 10-mL centrifuge tube, whereas the lower water phase was re-extracted with 4 mL Tris-saturated phenol (pH > 8.0). Phenolic phases was combined and precipitated overnight with 8 mL of 0.1 M ammonium acetate/methanol at −20 °C. After it was successively rinsed in 5 mL ice-cold 100% acetone and 80% acetone, the pellet was transferred to a 2-mL microtube and rinsed twice in 1 mL ice-cold 100% acetone. The final pellet was air-dried for 1.5 h at room temperature and dissolved in lysis buffer (8 M urea, 2 M thiourea, 4% 3-[(3-Cholamidopropyl)dimethylammonio]propanesulfonate, 1% dl-Dithiothreitol, 0.5% pH 3–10 non-linear gradient (NL) immobilized pH gradient (IPG) buffer, and a trace of bromophenol) for 1.5 h at room temperature. The protein solutions were centrifuged at 16,100× *g* for 40 min at 4 °C. The protein concentration of the supernatants was determined using bovine serum albumin (BSA) as a standard according to the Bradford method [58].

### 4.4. 2-DE and Staining

A sample containing 1350 μg proteins was loaded onto a 24-cm pH 3–10 NL IPG strip (GE Healthcare, Princ-eton, NJ, USA), which was rehydrated for 16 h at 20 °C. After rehydration, isoelectric focusing (IEF) was performed in a PROTEAN IEF system (GE Healthcare, Fairfield, CT, USA) under the following conditions: a gradient from 0 to 100 V for 4 h, 250 V for 1 h, 1 kV for 1 h, a gradient from 1 to 10 kV for 2 h, and a gradient from 10 to 100 kV for 12 h. Subsequently, the strip was equilibrated for two periods of 15 min with 1.0% (*w*/*v*) DTT and 2.5% (*w*/*v*) indole-3-acetic acid (IAA) in equilibration buffer (50 mM Tris-HCl pH 8.8, 6 M urea, 30% (*v*/*v*) glycerol, 2% (*v*/*v*) SDS, and a trace of bromophenol). SDS-PAGE was performed on vertical 12% SDS-PAGE self-cast gels with an Ettan DALTsix System (GE Healthcare, Fairfield, CT, USA) under the following conditions: 1 W for 30 min and 15 W for 6 h at 15 °C. After 2-DE, the gel was stained with 0.12% Coomassie brilliant blue (CBB) G-250. At least three biological replicates were assessed for each time point.

### 4.5. Image Acquisition and Statistical Analysis

The stained gels were scanned with an Image Scanner III (GE Healthcare, Fairfield, CT, USA) with default parameters as follows: optical resolution, 300 dots per inch (dpi); brightness, 3; contrast, −9; saturation, 9; total input value, 140; and output value, 20. These images were analyzed on PDQuest V 8.0 (Bio-Rad, Hercules, CA, USA). After the images were properly cropped and optimized, spot detection and gel-to-gel matching were performed automatically and were refined by manual spot editing when needed. Three well-separated gels for each time point were used to create “replicated groups”. We only considered “consistent spots”, which were present in the three biological replicates, thus preventing the assignment of normalized volume values to missing spots for multivariate analysis. These consistent spots were added to the master gel so they could be matched to all of the samples. The experimental molecular weight (MW) and isoelectric point (pI) of proteins were estimated using their position in the 2-DE gel.

All data regarding the protein spots from 2-DE maps were preprocessed according to the recommendations proposed by Valledor and Jorrin [59]. The protein abundance of each spot was normalized as a percentage of the total volume of all the spots present in the gel, to correct for variability due to quantitative variations in the intensity of the protein spots. Differentially regulated spots were defined with one-way ANOVA using SPSS v. 13.0 (Available online: http://spss.en.softonic.com/). Spot values passed the Duncan test and the degree of freedom (DF) was 11. False discovery rate (FDR) was controlled at level 0.05. A multivariate analysis was performed over the whole set of spots and on those showing differences. Spots whose regulation intensity was more than 1.5 times (*w*/*v* 0.05) or less than 0.67 times (*p* < 0.05) that of the control (0 h) at one or more time points were considered as differentially regulated protein spots for further analysis.

### 4.6. Protein In-Gel Digestion and Identification by MALDI-TOF/TOF-MS

Protein spots of interest were excised from the gels, transferred into sterilized 2-mL microtubes, and then washed twice with double-distilled water. Protein spots were repeatedly de-stained using 50 mM ammonium bicarbonate in 50% (*v*/*v*) acetonitrile (ACN) for 30 min at 37 °C. Subsequently, the gel pieces were shrunk by dehydration in ACN and then swollen for 30 min at 4 °C in digestion buffer (25 mM ammonium bicarbonate, 10% (*v*/*v*) ACN and 0.02 μg/mL trypsin). After digestion for 16 h at 37 °C, the supernatants were collected and the peptides were extracted again using 0.1% (*v*/*v*) trifluoroacetic acid (TFA) in 67% (*v*/*v*) ACN for 30 min. The two supernatants were combined, vacuum-dried, and then re-dissolved in 67% (*v*/*v*) ACN and 0.1% (*v*/*v*) TFA for MS analysis.

Peptide mass determinations were performed using an ABI 4800 Plus MALDI-TOF/TOF Analyzer (Applied Biosystems, Foster City, CA, USA). Identification of the protein sample was conducted using Mascot Version 2.1 software (Matrix Science, London, UK) with the following optimized parameters: present in the National Center for Biotechnology Information (NCBI) nonredundant (nr) database, a member of the Viridiplantae taxon, a maximum of one missed cleavage, a fixed modification of carbamidomethyl (C), variable modifications of acetyl (protein N-term) and oxidation (C), a peptide mass tolerance of 100 ppm, and an MS/MS tolerance of 0.4 Da. The score threshold was greater than 50 (*p* < 0.05). If the peptides were matched to multiple members of a protein family or if a protein appeared under different names and accession numbers, only significant hits with the highest protein score were accepted for identification of the protein sample. When the values of two scores were very close, we took the reference of the experimental MW and pI.

### 4.7. Functional Analysis

The bioinformatics data of the successfully identified proteins were gained by GO and KEGG annotation. GO (Available online: http://www.geneontology.org) and KEGG pathway (Available online: http://www.genome.jp/kegg/pathway.html) analyses were performed with the PartiGene program (Available online: http://www.nematodes.org/bioinformatics/annot8r/index.shtml). Annot8r assigns KEGG (gene) pathways and GO (protein) terms based on BLASTX similarity (E-value < 1.0 × 10^−5^) and known GO annotations. The results of GO analyses are summarized in three independent categories (biological process, cellular component, and molecular function).

### 4.8. Antioxidant Enzyme Activity Assays and Lipid Peroxidation Analysis

*Phalaenopsis* petals (0.5 g) were ground in ice-cold 100 mM phosphate buffer (pH 7.5) on ice. Homogenates was centrifuged at 1500× *g* for 20 min at 4 °C. The supernatant was used for subsequent assays. Superoxide dismutase activity (SOD; EC 1.15.1.1), catalase activity (CAT; EC 1.11.1.6), and peroxidase activity (POD; EC 1.11.1.7) were measured according to Chakrabarty et al. [51]. The measurement of lipid peroxidation, which is determined by measuring malondialdehyde (MDA), was assessed as described previously [11]. CAT and POD activity was expressed as U·g fresh weight (FW)^−1^. SOD activity and MDA content were expressed as U·min^−1^·g FW^−1^ and nmol·g FW^−1^, respectively.

### 4.9. Statistical Analysis of Physiological-Biochemical Changes

The significance of the differences between the pollination treatment (8, 16, and 24 h) and the control (0 h) were determined with one-way ANOVA test (*p* < 0.05) using SPSS v. 13.0 package (Available online: http://spss.en.softonic.com/). A repeated measurement is given as the mean ± SD.

## 5. Conclusions

Senescence is a very complex process that involves changes at the physiological, biochemical, and molecular biology levels. To explore the mechanism underlying senescence, we performed a comparative proteomic analysis combining several approaches, including ultrastructural observation and antioxidant enzyme activity analysis, on *Phalaenopsis* petals at 0, 8, 16, and 24 h after pollination. The petals appeared to be visibly wilting 24 h after pollination, and this could be accompanied by the mass degradation of macromolecules and organelles (Figure 1). Proteomic analysis yielded 42 differentially regulated proteins, including 17 proteins that were upregulated and 25 proteins that were downregulated, and these were identified with confidence by MALDI-TOF/TOF-MS and homology-driven searches. These protein species are likely to be involved in a wide range of cellular pathways. Taken together, the results suggest that multiple cellular pathways operate in a coordinated manner during petal senescence. The identified protein species with specific expression patterns can be used as putative markers of senescence. Additionally, the activity of all of the ROS-scavenging enzymes increased, keeping the ROS content at a controlled level at the early stage of senescence. In summary, the 2-DE proteomic data, ultrastructural observations, and physiological-biochemical analysis results presented here will help us further understand the molecular and biochemical changes that occur during petal senescence, thus providing a basis for prolonging florescence. However, as to the progress of senescence, additional studies will be necessary to fully elucidate the complexity of this process.

## Figures and Tables

**Table 1:** Protein yield (mg/g fresh weight), number of spots, and significant quantitative difference (spots up/downregulated) in *Phalaenopsis* petals at each time point.

Samples	Protein Yield	Number of Spots	Quantitative Difference	
	(mg/g Fresh Weight)	(Mean ± SD)	Number of Upregulated	Number of Downregulated
0 h	6.03 ± 0.94	1069 ± 92	0	0
8 h		1061 ± 203	1	14
16 h		1056 ± 80	5	22
24 h		1014 ± 8	16	23

**Table 2:** Identification, functional categorization, and quantification of the differentially regulated protein species in *Phalaenopsis* petal during pollination-induced senescence.

Spot No. ^a^	Protein Name and Organism ^b^	Accession No. ^c^	Exp./Theo. ^d^		Score/Matched Peptides/Coverage ^e^	Cellular Location ^f^	False Discovered Rate ^g^	Changes of Regulation Intensity ^h^
			**MW (kDa)**	**pI**				
**Antioxidation**								
10	Putative thioredoxin-dependent peroxidase (*Elaeis guineensis*)	gi|448872680	30.49/23.63	5.48/6.61	587/6(6)/40	V/ER/GA/C/M	0.002	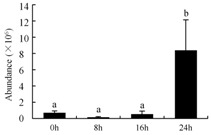
7	Thioredoxin H-type (*Zea mays*)	gi|195645418	6.03/14.06	5.39/5.27	359/5(4)/37	ER	0.046	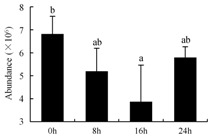
14	Glutathione *S*-transferase (*Medicago truncatula*)	gi|357460737	24.93/32.54	5.62/6.52	259/3(1)/10	CW/N/C/M/GA/Pl/Ch/A	0.000	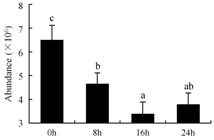
16	Glutathione S-transferase (*Vitis amurensis*)	gi|224038272	24.85/32.54	5.95/6.52	325/4(2)/14	C	0.003	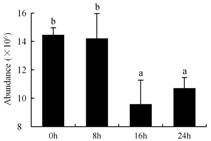
34	Glutathione S-transferase (*Triticum urartu*)	gi|474401794	25.72/29.98	7.61/5.51	326/3(3)/17	C/A	0.011	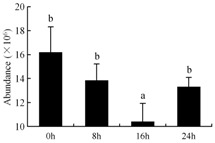
41	Nicotinamide adenine dinucleotide (phosphate)(NAD(P))-linked oxidoreductase superfamily protein (*Theobroma cacao*)	gi|590655852	54.05/16.20	7.68/8.44	54/1(1)/9	C	0.005	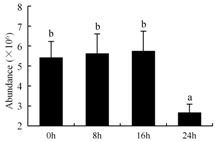
**Stress Response**								
20	17.7 kDa heat shock protein (*Carica papaya*)	gi|37933812	14.37/24.02	6.33/5.26	448/3(3)/15	N/ER	0.000	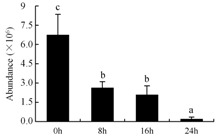
29	Heat shock protein 17.9 (*Cenchrus americanus*)	gi|238915387	16.30/19.00	7.23/9.30	535/5(3)/21	N/C/M/A	0.002	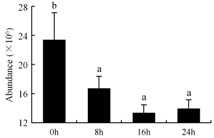
30	17.4 kDa heat shock protein (*Oryza sativa* Japonica group)	gi|313575791	16.30/25.37	7.23/5.57	654/6(5)/18	-	0.000	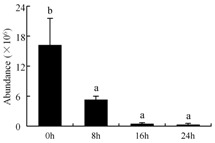
33	Small molecular heat shock protein 17.5 (*Nelumbo nucifera*)	gi|118452817	13.31/17.57	7.42/5.94	147/2(1)/10	-	0.025	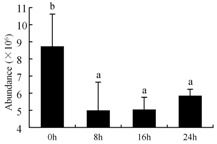
5	Heat shock protein Hsp70 (*Mucilaginibacter paludis*)	gi|495787168	30.52/25.82	5.01/5.57	346/3(3)/15	Mi/C/Ch/A	0.000	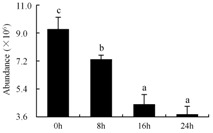
12	Heat shock protein 70 cognate (*Populus trichocarpa*)	gi|224100969	62.31/71.53	5.49/5.11	868/8(7)/19	Ch	0.005	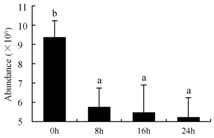
31	Phospholipase D (*Coffea arabica*)	gi|332182725	26.51/19.07	7.02/5.39	238/3(1)/27	-	0.015	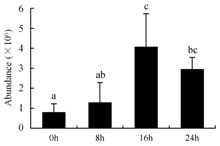
1	Lectin (*Cymbidium hybrid cultivar*)	gi|436827	6.68/13.26	4.55/9.42	171/2(2)/11	-	0.001	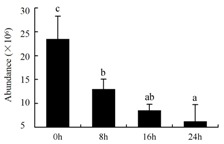
6	Dehydrin (*Hyacinthus orientalis*)	gi|47026904	39.03/19.30	5.13/6.37	173/2(2)/9	-	0.003	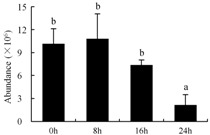
35	Dehydrin 13 (*Zea mays*)	gi|226501978	17.18/21.10	7.38/6.29	112/2(0)/9	-	0.003	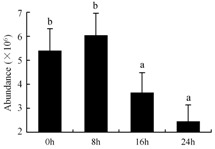
42	Alcohol dehydrogenase (NADP+) A (*Aegilops tauschii*)	gi|475594485	34.95/29.47	7.98/9.54	421/6(4)/14	AC	0.002	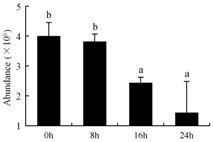
**Protein Metabolism**								
32	Cysteine proteinase (*Phalaenopsis* sp. *SM9108*)	gi|1173630	40.95/40.49	7.27/6.23	519/7(2)/23	C	0.043	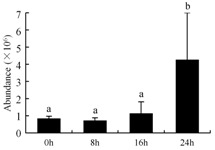
37	Ubiquitin fusion degradation protein 1, partial (*Solanum nigrum*)	gi|321149977	36.92/26.18	7.50/8.74	236/3(2)/16	C	0.001	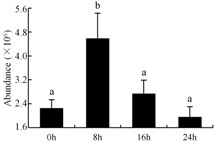
43	Proteasome subunit β type 1 (*Zea mays*)	gi|226531171	56.66/23.19	8.01/6.11	346/3(3)/19	Pe/N/C/M/Ch/A	0.048	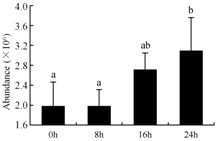
23	Aminopeptidase N (*Morus notabilis*)	gi|587846889	90.12/32.60	6.21/8.57	94/1(1)/4	-	0.007	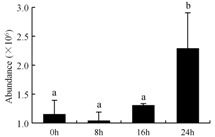
24	FK506-binding protein 2-2 (*Aegilops tauschii*)	gi|475591369	10.60/12.41	6.47/5.51	104/2(2)/16	Ch/P	0.000	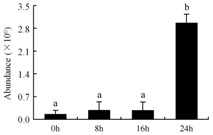
**Cell Wall Component Metabolism**								
9	Cellulose synthase-3 (*Zea mays*)	gi|9622878	30.25/25.82	5.16/5.57	335/3(3)/17	-	0.001	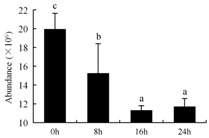
38	Xyloglucan endotransglucosylase/hydrolase (*Gossypium hirsutum*)	gi|308229784	32.37/31.07	7.32/9.16	144/1(1)/26	C	0.043	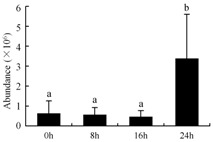
39	Cinnamyl alcohol dehydrogenase (*Lolium perenne*)	gi|19849246	44.18/24.10	7.59/8.77	204/2(2)/13	CW/Mi/V/C/Pl/Ch/A/M/GA	0.008	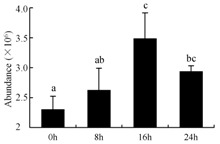
**Energy Metabolism**								
40	Pyrophosphate-fructose 6-phosphate 1-phosphotransferase subunit β (*Medicago truncatula*)	gi|357480393	55.94/62.89	7.47/5.88	376/4(3)/16	GA/M	0.004	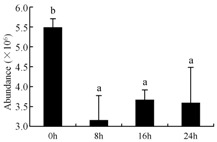
8	Triosephosphate isomerase (*Oryza coarctata*)	gi|165973012	25.24/20.21	5.22/5.19	434/4(4)/34	Mi	0.000	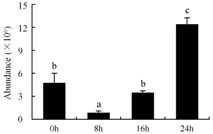
15	Triosephosphate isomerase (*Gossypium hirsutum*)	gi|295687231	26.74/33.50	5.61/6.66	230/4(3)/13	CW/Mi/M/Ch/GA	0.024	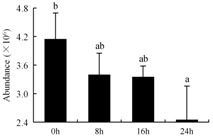
11	Dihydrolipoamide acetyltransferase component of pyruvate dehydrogenase (*Cucumis melo* subsp. *Melo*)	gi|307135863	54.07/27.53	5.51/7.08	183/2(2)/12	N/Mi/V/C/Pl/Ch/M	0.011	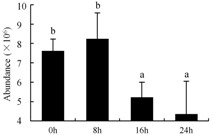
19	Dihydrolipoamide dehydrogenase (*Gossypium hirsutum*)	gi|211906492	15.06/28.79	6.15/5.47	300/4(3)/26	-	0.000	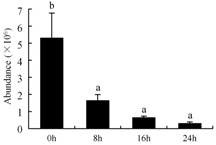
17	Pyruvate orthophosphate dikinase (*Eleocharis vivipara*)	gi|2285879	86.71/94.27	5.89/5.29	422/7(3)/17	N/C/Ch	0.042	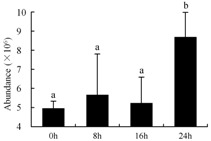
18	Pyruvate orthophosphate dikinase (*Amaranthus hypochondriacus*)	gi|336020527	88.12/96.65	5.72/5.21	504/8(5)/17	CW/A	0.037	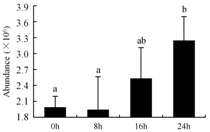
26	Mitochondrial adenosine triphosphate(ATP) synthase 24 kDa subunit, partial (*Oryza sativa* indica group)	gi|149392623	27.10/20.84	6.51/9.45	377/5(4)/16	C	0.003	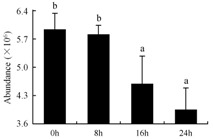
**Cell Structure**								
2	Putative profilin (*Phalaenopsis hybrid cultivar*)	gi|4512111	5.63/24.42	4.43/5.08	158/3(3)/19	C	0.000	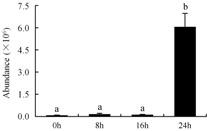
13	Actin-depolymerizing factor (*Gossypium barbadense*)	gi|161779424	10.83/33.22	5.69/8.89	435/4(4)/25	N/C	0.000	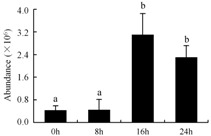
47	Myosin-like protein (*Zea mays*)	gi|195622168	28.79/25.88	8.54/9.23	237/2(2)/7	N/C/M	0.027	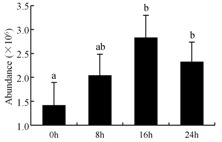
**Signal Transduction**								
25	14-3-3 protein (*Ipomoea nil*)	gi|124484407	19.03/29.64	6.72/4.76	415/5(3)/22	-	0.001	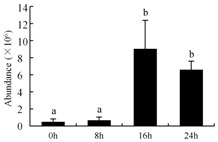
48	Annexin (*Populus tomentosa*)	gi|429326382	35.49/35.47	8.95/6.82	150/2(2)/8	N/C	0.004	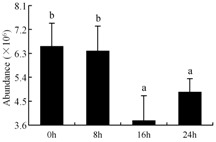
4	Putative guanosine triphosphate (GTP)-binding protein (*Leucocoprinus fragilissimus*)	gi|110349697	38.14/16.89	6.75/4.94	530/8(5)/27	Mi/A/GA	0.002	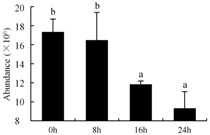
36	Casein kinase II subunit β-4 (*Zea mays*)	gi|195628750	31.06/26.71	7.74/8.19	114/2(1)/7	CW/C/M	0.001	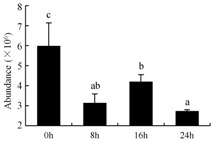
**Unclassified**								
44	Fiber protein, partial (*Hyacinthus orientalis*)	gi|42565482	5.89/16.53	8.82/5.93	202/2(2)/17	N/Mi/Ch/M	0.021	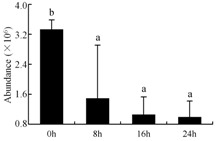
46	Mitochondrial outer membrane porin (*Triticum urartu*)	gi|473968092	26.76/37.68	8.75/9.25	738/7(7)/28	C/M/Ch	0.028	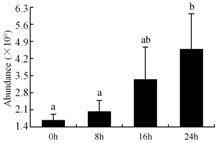

^a^ Spot No. corresponds to the spot in Figure 2; ^b,c^ Data of protein name and organism and accession no. are from the National Center for Biotechnology Information (NCBI) database of the matched protein species; ^d^ Experimental molecular weight (MW)/isoelectric point (pI) was calculated by Image PDQuest 8.0 software (Bio-Rad, Munich, Germany) according to position of protein spot in 2-D gel; theoretical MW/pI was gained from the matched protein species; ^e^ Score from MALDI-TOF/TOF-MS analysis for the most significant hits (*p* < 0.05); matched peptides indicates total number of peptides that matched to other protein species; coverage refers to percentage of matched protein species; ^f^ Data of cellular location are from Gene Ontology (GO) annotations (cellular component). A, apoplast; AC, actin cytoskeleton; C, cytosol; Ch, chloroplast; CW, cell wall; GA, Golgi apparatus; ER, endoplasmic reticulum; M, membrane; P, plastid; Pe, peroxisome; Pl, plasmodesma; V, vacuole; ^g^ Spot values passed the Duncan test, and false discovered rate (FDR) was controlled at level 0.05; ^h^ From left to right, each bar indicates the change in protein spot volume after pollination for 0, 8, 16, and 24 h. Spot values are presented as means ± SD of three replicates. Means distinguished with different letters are significantly different from each other (*p* ≤ 0.05).
